# Iron-fortified water: a new approach for reducing iron deficiency anemia in resource-constrained settings

**DOI:** 10.1038/s41598-023-40600-z

**Published:** 2023-08-21

**Authors:** Chicgoua Noubactep, Joseline Flore Kenmogne-Tchidjo, Sebastian Vollmer

**Affiliations:** 1 Centre for Modern Indian Studies (CeMIS), Universität Göttingen, Waldweg 26, 37073 Göttingen, Germany; 2 Department of Chemistry, Faculty of Sciences, University of Yaoundé I, P.O. Box 812, Yaoundé, Cameroon; 3 Department of Water and Environmental Science and Engineering, Nelson Mandela African Institution of Science and Technology, P.O. Box 447, Arusha, Tanzania; 4 Faculty of Science and Technology, Campus of Banekane, Université des Montagnes, P.O. Box 208, Bangangté, Cameroon; 5 School of Earth Science and Engineering, Hohai University, Fo Cheng Xi Road 8, Nanjing, 211100 China

**Keywords:** Biochemistry, Environmental sciences, Environmental social sciences, Health care, Chemistry, Engineering

## Abstract

A new approach for fortification of drinking water is presented for combating iron deficiency anemia (IDA) worldwide. The idea is to leach Fe from a bed containing granular metallic iron (Fe^0^), primarily using ascorbic acid (AA). AA forms very stable and bioavailable complexes with ferrous iron (Fe^II^). Calculated amounts of the Fe^II^-AA solution can be added daily to the drinking water of households or day-care centers for children and adults (e.g. hospitals, kindergartens/schools, refugee camps) to cover the Fe needs of the populations. Granular Fe^0^ (e.g., sponge iron) in filters is regarded as a locally available Fe carrier in low-income settings, and, AA is also considered to be affordable in low-income countries. The primary idea of this concept is to stabilize Fe^II^ from the Fe^0^ filter by using an appropriate AA solution. An experiment showed that up to 12 mg Fe can be daily leached from 1.0 g of a commercial sponge iron using a 2 mM AA solution. Fe fortification of safe drinking water is a practicable, affordable and efficient method for reducing IDA in low-income communities.

## Introduction

Iron deficiency is reported to be the most prevalent nutritional deficiency worldwide, affecting about 5 billion people^1–4^. Iron deficiency is the main cause of anemia (iron deficiency anemia—IDA) in children and women^4,5^. The prevalence of anemia is five times higher in low-income countries than in high-income countries^6,7^. Another important reason for this high prevalence is that infectious diseases such as bacterial infections, hookworms, human immunodeficiency virus or acquired immunodeficiency syndrome (HIV/AIDS), intestinal parasitic infections, malaria, and tuberculosis are common conditions associated with a reduction of blood constituents, including iron^1,2,4^. Iron deficiency impairs cognitive development in children, reduces work capacity in adults, increases the risk of morbidity, induces poor pregnancy outcomes, and impairs immunity^1,3,4,8^. Iron deficiency is considered to be one of the most expensive diseases in the world, according to the World Health Organization (WHO)^3,4,8–11^. This is because IDA induces economic losses in the form of (1) cognitive losses among children, (2) lower productivity among adults, and (3) associated increased medical expenses for all groups^9,11^. Fortunately, IDA is preventable and curable, and doing so is a priority for the WHO^10,11^. However, despite concerted efforts for iron supplementation (e.g. beverages, food fortification, injections, syrups, drugs, pills, salt fortification, tablets), not much progress has been realized in the past three decades^3,4,7,12–16^. IDA maintains its top position as the leading cause of years lived with disability, and still contribute to up to 120,000 maternal deaths per year^3,11,17^.

Many strategies to prevent and treat IDA have been developed over the years^2,4,7,12,18–20^. Table 1 gives an overview of the used tools based on the oxidation-state-dependent characteristics of the used iron sources (Fe^0^, Fe^II^, and Fe^III^). The four most commonly used strategies are: (1) dietary diversification, (2) food fortification with Fe, (3) multi-micronutrient supplementation, and (4) treatment of parasitic infections contributing to iron deficiency^2,3,5^. A fifth strategy that has been reported only in Brazil, is drinking water fortification with soluble ferrous iron (Fe^II^)^7,12,21–24^. In this effort, a favored short-term and cost-effective strategy that is relatively easy to implement at large scale, is the use of fortification programs at kindergartens and schools^5^. For the prevention of IDA, the WHO recommends a daily iron supplementation for three consecutive months in a year to all pre-menopausal women, adolescent girls and young children in the majority of countries^3,11^.

**Table 1:** Characteristics of the three main sources of iron for controlling iron deficiency anemia.

OS (−)	Iron source	Solubility	RBA (%)	Comments
0	Iron powders	Very low soluble	75	Unknown corrosion
0	Iron ingots	Very low soluble	n.a	Unknown corrosion
II	Fe(II) sulfate	Highly soluble	100	Least expensive
II	Fe(II) chloride	Highly soluble	50	Not stable in air
III	Fe(III) ammonium citrate	soluble	51	Soluble, not bioavailable
III	Fe(III) pyrophosphate	Very low soluble	43	Unknown dissolution, not bioavailable

The most readily available iron sources are selected with two per state of oxidation (oxidation state—OS). RBA stands for the relative bioavailability, RBA is related to the solubility in 0.1 N HCl (Harrison et al. (1976) cited by Kumari and Chauhan^4^).

The major challenge in combating IDA is the control of the iron bioavailability^7^. Iron sources are widely available on the market but not all of them are bioavailable (Table 1). Table 1 shows that the best iron source (bioavailable) is Fe^II^ sulfate with stable Fe^II^. Fe^II^ chlorides are also highly water soluble, but not stable under physiological conditions. Fe^0^ and Fe^III^ sources are less soluble and their bioavailability is correspondingly low. In other words, soluble Fe should be present as stable Fe^II^ to be absorbed by the human body^2,7,17,25^. This is the reason why the simultaneous consumption of Fe rich diets and vitamin C (ascorbic acid—AA) has consistently and successfully treated IDA^2,7,22,24,26^. Ascorbic acid is a strong reducing agent for Fe^III^ and forms very stable complexes with Fe^II^ [Fe^II^-AA]^27–29^. Fe^II^-AA complexes are readily absorbed by the human body. Thus, supplying people with Fe^II^-AA solutions has the potential to decrease IDA^7,12^.

This communication presents the design of a device to locally generate Fe^II^-AA solution for drinking water fortification. Granular Fe^0^ is placed in a column and leached with a solution of AA. A calculated amount of the leachate is added to the drinking water tank to yield the daily needed iron concentration. Tools for the implementation of such devices in day-care centers for children and adults are discussed.

The presentation starts with a description of the Fe/AA system ("Background of the Fe^II^-AA concept" section), followed by a discussion of current uses of Fe^0^ for IDA control ("Current use of Fe^0^ for IDA control" section). "Fe^0^ leaching with ascorbic acid: proof of concept" section presents some experimental data on Fe^0^ leaching with ascorbic acid (e.g. proof of concept), "Designing a Fe^II^-bearing unit" section prepares the realization of the concept by discussing a Fe^II^-AA production unit. "IDA and safe drinking water provision: killing two birds with one stone?" section formulates some recommendations for combining IDA control and safe drinking water provision. A short conclusion ("Concluding remarks" section) closes the presentation.

## Background of the Fe^II^-AA concept

The ability to improve iron status in populations largely depends on the understanding of the biochemistry and absorption of Fe in the human body^2,7,17,28^. There are two types of iron: heme (found in red meat) and non-heme (found in plant-based foods) iron. Iron absorption in the gastrointestinal tract is lower for non-heme sources of iron. The literature contains many contradictory findings regarding parameters pertinent to the effective absorption of Fe by humans^3,5,17^. It seems established that ferrous salts are better than heme iron in combatting IDA, however, some newer iron formulations have claimed the opposite^30,31^. Fortunately, it is unequivocally reported that a combination of ascorbic acid (AA) and Fe-bearing diets improves the iron status in populations^7,30^. Being a weak acid, AA is a strong reducing agent for Fe^III^ and an excellent complexing agent for Fe^II^^7,28,29^. This means that, where necessary, AA reduces aqueous Fe^III^–Fe^II^ and builds the very stable Fe-AA complex which is available for the human body (Fact 1: AA reduces aqueous Fe^III^ and builds stable Fe-AA complex). Therefore, Fe-AA complexes are bio-available in the human body^7^.

People living in a high-iron groundwater setting have demonstrated better iron status or suffer less from IDA^32–38^. The rationale for this finding is that Fe-rich groundwater contains Fe^II^ which is oxidized to Fe^III^ upon contact with air (20% O_2_)^39^. This implies that the amount of bioavailable Fe^II^ also depends on the duration of storage. Upon oxidation Fe^III^ precipitates as Fe hydroxides/oxides or is complexed to less/non bioavailable species. Fe-rich groundwater contains bioavailable Fe^II^. Whenever clean Fe-rich groundwater is available as a drinking water source, it suffices to stabilize Fe^II^, for example by addition of AA or lemon juice, to improve the Fe status of the population^25,40,41^.

Taken together, supplying populations with drinking water containing Fe stabilized in the ferrous form (Fe^II^) is sufficient (Fact 1). This idea is not new, as it has been successfully applied in rural Brazil for the last three decades^7,21–24,26^. In its original form, each family was required to have an earthen pot with about 10 L capacity for storing drinking water. Families received a concentrated iron solution (l0 g/L) in the form of ferrous sulfate (FeSO_4_) and L-ascorbic acid in the molar ratio Fe^II^:AA = 1:3, dispensed in l0 mL bottles^21^. The success of this approach has motivated its extension to day-care institutions^7,24^. The present work seeks to leach Fe^II^ from metallic iron (Fe^0^). Thus, commercial ferrous sulfate is replaced by a more affordable granular Fe^0^, which is additionally readily available, for example as iron filings, iron wire, scrap iron, sponge iron (direct reduced iron), or steel wool. Fe^0^ sources to be considered in this context should not contain any toxic alloying elements. Sponge iron is certainly the best material fulfilling this prerequisite. The typical mineralogical composition (in %) of sponge iron is^42,43^: Fe (total): 92–95; Fe^0^: 85–90; C: 1.0–1.5; S: 0.005–0.015; P: 0.02–0.09, SiO_2_: 1.0–2.0, and balanced by the gange. The gange is the residual unreduced oxides, mainly comprising of Al_2_O_3_, CaO, FeO, MgO, MnO, and SiO_2_. The typical mass density of sponge iron is 1600 kg m^−3^ and its apparent density is 3200 kg m^−3^^42^.

Materials selected herein for use in the Fe fortification unit are known to be effective for producing stable soluble Fe^II^ solutions under environmental conditions. Availability and cost are also considered in the selection process because substantial quantities would be required for decentralized production. Used Fe^0^ particles should not contain toxic alloying elements (e.g. Cr, Ni). Fortunately, this is the case for many readily available Fe^0^ materials such as cast iron and low alloyed steel. For example, Lufingo et al.^29^ analyzed nine commercial steel wool for their elemental composition and found that the Fe^0^ content was constantly higher than 99%, while the (Cr + Ni) level was lower than 0.7% in all the specimens. These data suggest that a solution containing some 10 mg/L of Fe would contain non-detectable levels of (Cr + Ni). However, ideally, used Fe^0^ specimens should be free of Cr, Ni and Pb. Therefore, there is a need to (1) determine the chemical composition of potential Fe^0^ materials, and (2) test them with regard to the leaching ability of relevant toxic elements before their use for drinking water fortification. The next section presents a proof of concept, limited to illustrating the Fe leaching capability of a 0.02 M AA solution (pH = 3.5) from four selected Fe^0^ specimens, in five parallel experiments.

## Current use of Fe^0^ for IDA control

**Figure 1:**
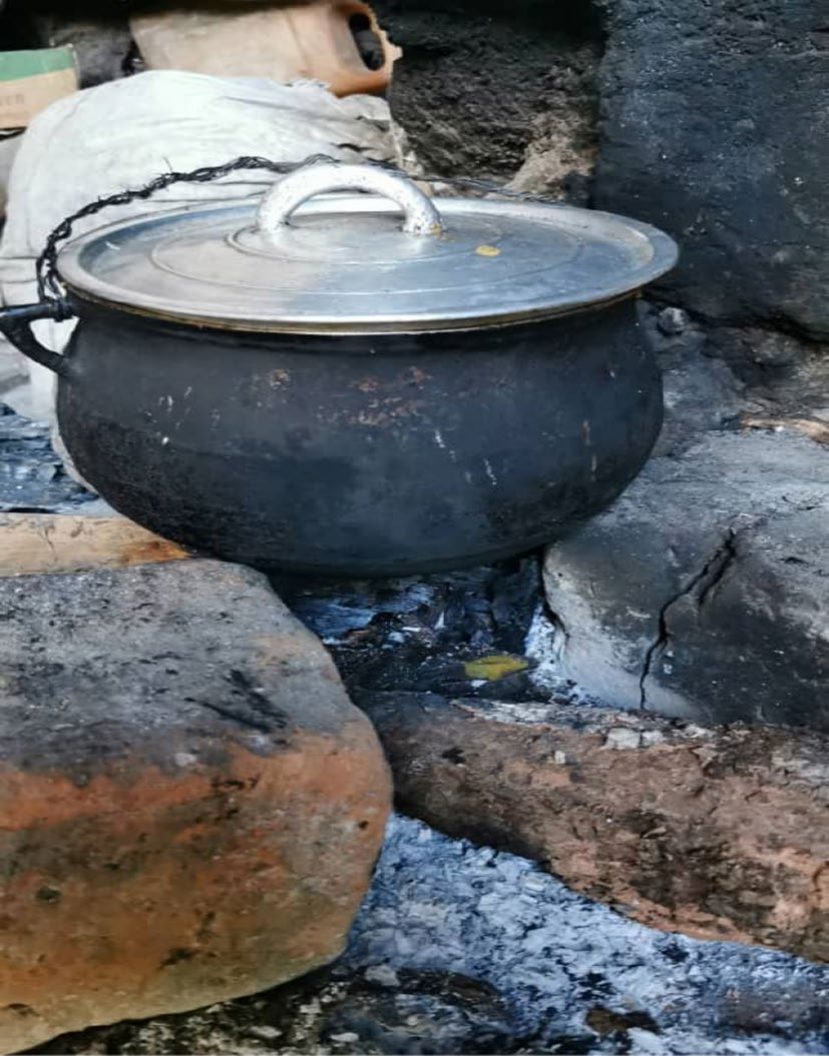
Photograph of an aluminum cooking pot on the fire in Bamena (rural Cameroon). Photograph taken by Serge Ndokou-Nana, October 2021.

**Figure 2:**
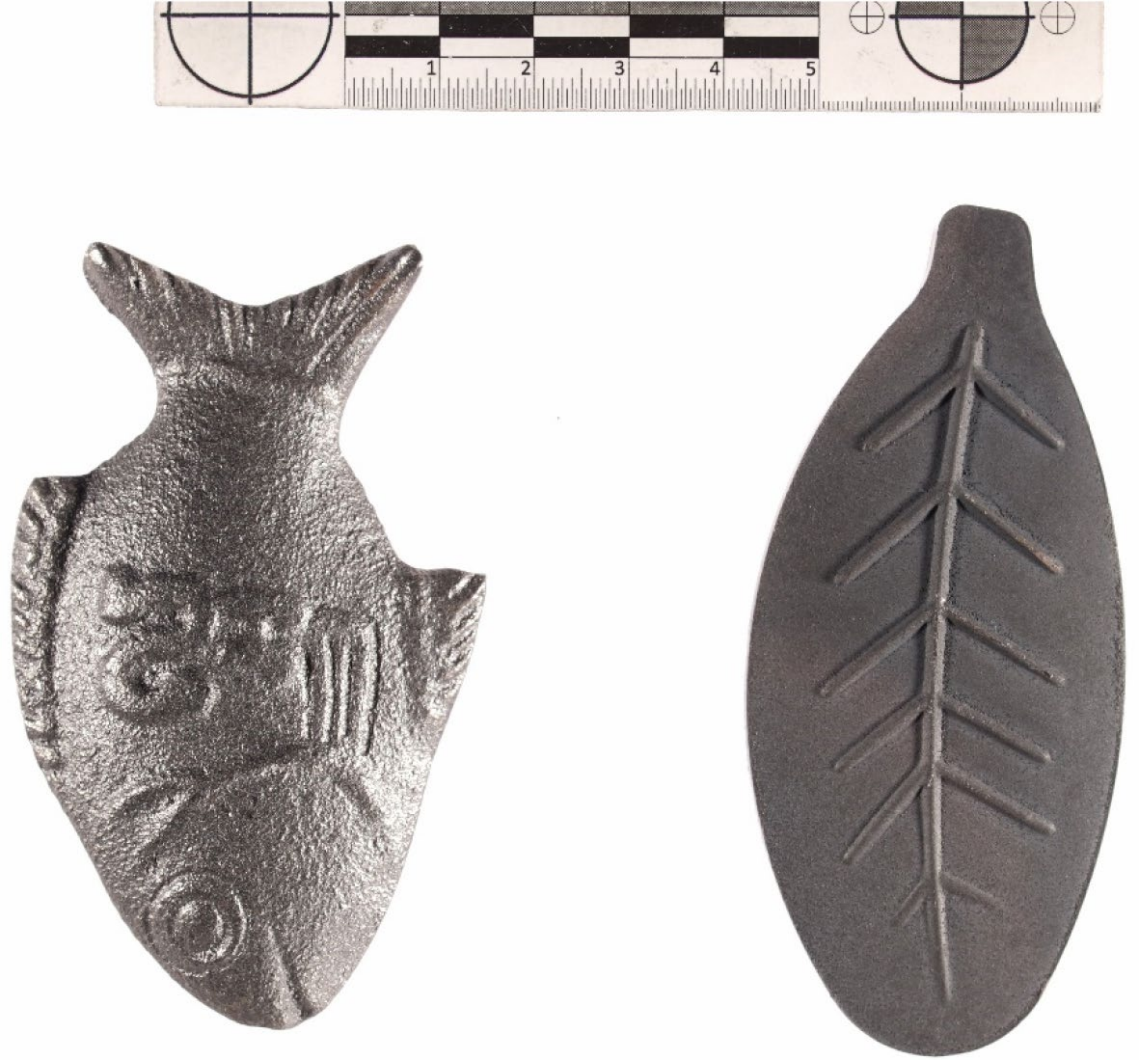
Photograph of a Lucky Iron Fish and a Lucky Iron Leaf. Photograph taken by Gerhard Hundertmark, November 2021.

Fe^0^ is currently considered an adventitious source of bioavailable iron with both adverse and beneficial effects on human health (Tables 1, 2)^44–50^. On the one hand, excessive Fe intake (e.g. Fe overload or iron poisoning) is attributed to metallic poisoning derived from foods and drinks prepared in Fe^0^-based vessels^2,18–20,44,45^. On the other hand, Fe leached from Fe^0^-based cooking utensils is recommended to prevent and cure IDA^18–20,40,49,50^. Where Fe^0^ cooking utensils are not available, not affordable, or not socially accepted, reusable Fe^0^ ingots have been used^51–55^, for example in Al-based cooking utensils (Fig. 1). Figure 2 shows the photograph of a fish-shaped iron ingot as used for in-situ food fortification in Cambodia as well as a leaf-shaped iron ingot as used in India^55,56^.

**Table 2:** Overview on the status of current approach to exploit metallic iron (Fe^0^) to controlling iron deficiency anemia (IDA).

Tool	Status	References
Leaching Fe from cooking pots	Field applications	^57^
Placing Fe^0^ in drinking water containers	Experimental	^38^
Leaching Fe from water pipes	Experimental	^38^
Placing Fe^0^ ingots in cooking pots	Field applications	^58^
Leaching Fe from Fe^0^ particles	Conceptual	This study

Fe^0^ in the form of iron powders has also been widely used in food fortification^14,17,56^. In this context, Fe absorption is governed by the extent of the dissolution of used Fe^0^ powders in the gastric fluid^17^. The extent of Fe absorption thus depends on the intrinsic reactivity of the used Fe^0^ in the human gastric fluid. The lack of characterization of the Fe^0^ intrinsic reactivity seems to be a major shortcoming as many different Fe^0^ types have been tested and used without appropriate quality control^58^. A proper quality control would characterize the relative Fe bioavailability from used Fe^0^ powders. For example, H-reduced Fe^0^ powders for fortifying cereal flours have been largely used, while the WHO recommends only electrolytic iron powder^14^. The WHO recommendation is based on field evidence from Fe absorption in efficacy studies. However, it would have been better to develop an operational parameter (e.g. a dissolution index) to assess the Fe^0^ dissolution trend under different physiological conditions (in the gastrointestinal tract).

While it is clear that Fe^0^ is a relevant Fe source to cure and/or prevent IDA, it is not clear why a specific Fe^0^ source is preferentially used, and which specific operational conditions are optimal to meet the daily needs of a human being^2,7,17,23^. Relevant Fe^0^ sources include: (1) Fe^0^ cooking and storing vessels (pots and drums)^44,45,59^, (2) reusable Fe^0^ ingots^51–53,55,60^ (https://luckyironfish.com, Access 2021/10/25), and (3) Fe^0^ powders^2,56,61,62^.

Food fortification is largely considered the best strategy to increase iron intake of a population, especially for children and pregnant women^2,14–16,55^. The WHO has recognized food fortification as a potential universal tool for defeating IDA worldwide^11^. However, there are several concerns to be named: (1) by solving one problem (IDA) in some people, universal fortification exacerbates Fe intoxication for other people (questioned universality), and iron poisoning is as severe as IDA^2^, (2) because of low income, a large fraction of the population has only restricted access to commercial fortified foods (questioned affordability), and (3) it is not known which fraction of Fe^0^ in food is effectively solubilized during digestion and which proportion is absorbed by the body of each individual person^2,7,17,23^.

With the objective of solving the three named problems this communication suggests a solution that is beneficial to the segment of the population (potentially) suffering from IDA. This solution is called ‘semi-universal’ fortification^2^ and uses water as a vehicle^7^. Moreover, only drinking water is fortified and is considered affordable or at least more affordable than commercial fortified foods. Concerning the bioavailability, Fe is leached from granular Fe^0^ by ascorbic acid and is long-term stable and bioavailable^7,21–24,26,28^.

## Fe^0^ leaching with ascorbic acid: proof of concept

### Fundamental aspects

The present study presents a concept to extract Fe^II^ from Fe^0^ specimens, using ascorbic acid (AA) as leaching agent or lixiviant. Previously, AA has been used to leach and extract metals from natural metal oxides (e.g. marine MnO_2_) by reductive dissolution^63–66^. In this context, AA is a reduction and leaching (chelating) agent for ore processing at ambient temperature and under normal pressure. A key lesson from this hydrometallurgical process is that AA leaching has good dynamic characteristics, high reaction kinetics, and requires simple equipment. In this paper, AA is used to sustain the oxidative dissolution of Fe^0^ specimens. Fe^0^ is oxidized by water (H^+^) (oxidative dissolution) (Eq. 1) and the resulting Fe^2+^ is stabilized by chelation with AA (Eq. 2). In the absence of AA, Fe^2+^ would have been further oxidized to Fe^3+^ by oxygen present in air (Eq. 3) and precipitated as Fe(OH)_3_ (Eq. 4)^58,67–70^. From Eq. (1), a tool to increase the extent of Fe^2+^ leaching is to lower the pH value (H^+^ addition).

1 $${\text{Fe}}^{0} + {\text{2 H}}^{ + } { \Rrightarrow } {\text{Fe}}^{{{2} + }} + {\text{H}}_{{2}}$$

2 $${\text{Fe}}^{{{2} + }} + {\text{AA}}{ \Rrightarrow } {\text{Fe}}\left( {{\text{AA}}} \right)^{{{2} + }}$$

3 $${\text{4 Fe}}^{{{2} + }} + {\text{O}}_{{2}} + {\text{2 H}}^{ + } { \Rrightarrow } {\text{4 Fe}}^{{{3} + }} + {\text{2 OH}}^{-}$$

4 $${\text{Fe}}^{{{3} + }} + {\text{3 OH}}^{-} { \Rrightarrow } {\text{Fe}}\left( {{\text{OH}}} \right)_{{3}}$$

Once Fe(AA)^2+^ complexes are formed (Eq. 2), they remain stable even when the pH increases to values as high as 8.0^71,72^. In particular, Conrad and Schade^71^ demonstrated that, adding NaOH to a (FeCl_3_ + AA) solution results in a soluble iron chelate, while adding AA to a (FeCl_3_ + NaOH) mixture results in an insoluble Fe(OH)_3_.

Organic acids (e.g. acetic acid, citric acid, oxalic acid) and other chelating agents (e.g. ethylenediaminetetraacetic acid—EDTA) can be used as effective lixiviants for fly ash and minerals^73,74^. Organic acid mixtures are currently tested to recover valuable metals from spent Li-batteries^75^. For example, the process described by Chen et al.^74^, used iminodiacetic acid and maleic acid to quantitatively recover Li^+^ and Co^3+^ at 60 °C. AA then converts Co^3+^–Co^2+^ and enables selective recovery of Co. The present work uses AA to sustain Fe^0^ dissolution (Eq. 1). Comparable approaches are efforts from our research group using two organic chelates (EDTA and 1,10-Phenanthrolin) to characterize the intrinsic reactivity of Fe^0^ specimens^29,58,76^. Moreover, our research group has been routinely using a 0.1 M AA as a washing solution to free glassware from Fe^III^ oxides after Fe^0^ decontamination experiments.

### Experimental procedure

This section is adapted from Ndé-Tchoupé et al.^76^ who characterized the reactivity of twelve Fe^0^ materials for H_2_ evolution in H_2_SO_4_. The four tested herein were included, and depicted significant different reactivity. This result was recently confirmed using a newly developed test for Fe^0^ screening: the ascorbic acid test^58^.

#### Solutions

The working solution was prepared from a L-ascorbic acid powder (Merk, Darmstadt, Germany). The used 1,10-Phenanthroline, sodium ascorbate, and the iron standard (1000 mgL^−1^) were also from Merck (Darmstadt, Germany). All chemicals were of analytical grade.

#### Iron materials

Four selected Fe^0^ materials were used. Two of them were commercially available materials for groundwater remediation termed as: (1) “sponge iron”, and (2) “iPuTec”. Sponge iron is Eisenschwamm from ISPAT GmbH, Hamburg; while iPuTec is Graugußeisengranulat from iPutec GmbH & Co. KG, Rheinfelden; both in Germany. The other two materials were scrap iron materials from a metal recycling company (Metallaufbereitung Zwickau) termed as: “S15” and “S69”. S15 was a mixture of mild steels from various origins, while S69 was a similar mixture of cast irons. Apart from sponge iron, Fe^0^ materials were used in their typical state and form (i.e., “as received” state). Sponge iron was crushed into small pieces, sieved and the particles with sizes ranging between 1.0 and 1.6 mm were used, without any further pre-treatment.

Table 2 summarizes the elemental compositions of the materials based on analyses made using X-Ray fluorescence spectrometry. It can be clearly seen that the materials primarily differ in their carbon (C) and silicon (Si) contents. Thus, based on the C content, the tested materials can be divided into three classes: (1) iPuTec and S69 containing more than 3% C (cast irons), (2) S15 containing less than 2% C (mild steel), and (3) sponge iron (1.9% C), belonging to the third class, characterized by a specific manufacturing technology, which yielded porous materials^42,43^. All these materials were irregular in shape (filings and shavings) with rough surfaces. Sponge iron had a very rough surface and was even porous. iPutec and the two scrap irons (S15 and S69) were visibly covered with rust.

#### Experimental methods

**Figure 3:**
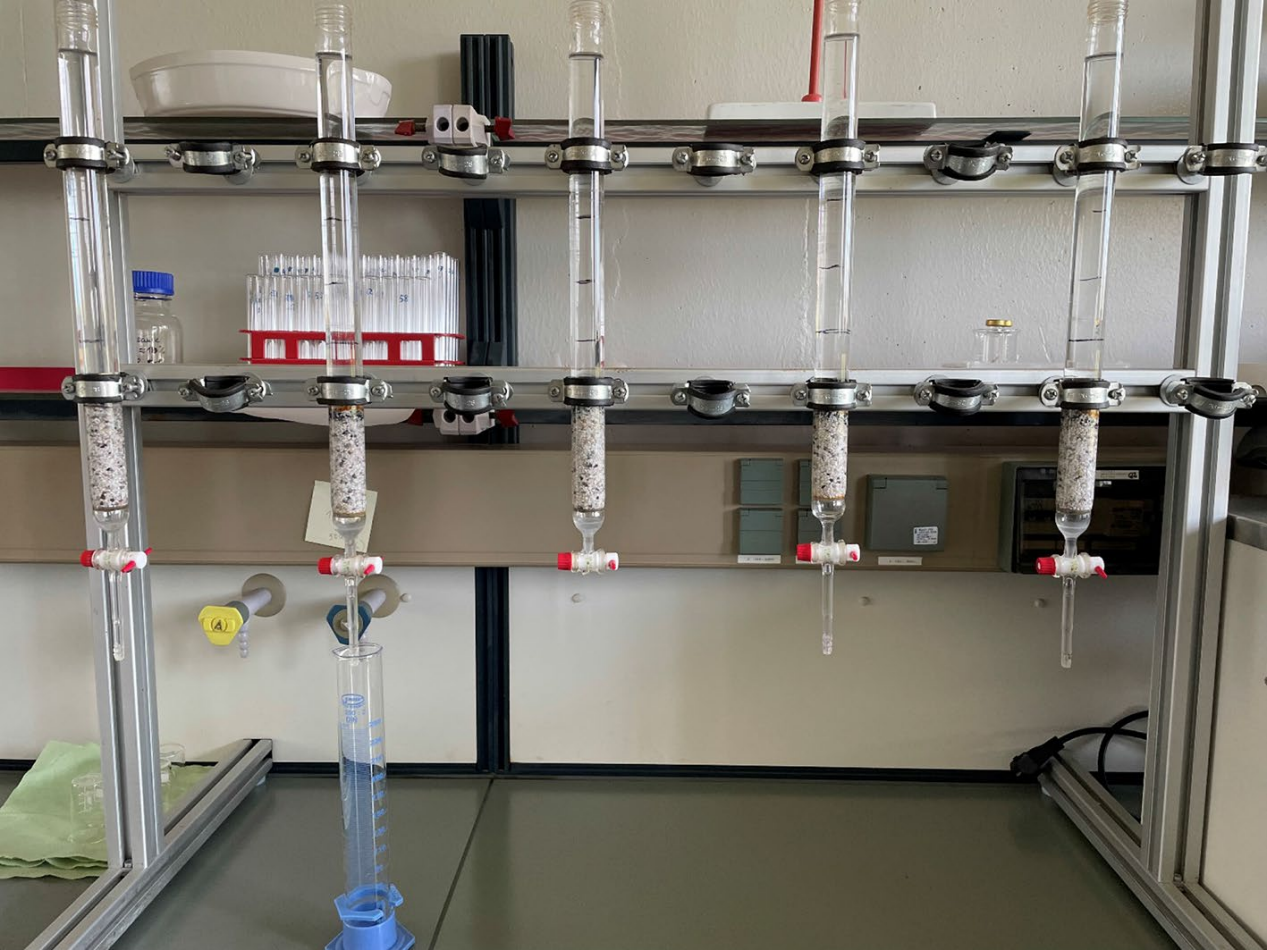
Column experimental set-up for Fe^0^ leaching by ascorbic acid (2 mM). The photograph was made at the end of the experiments. The spout of the third column was broken during the experiments but this has no incidence on the performance of the system.

1.0 g of each Fe^0^ material was placed in a chromatographic column containing sand in its lower third and the 0.02 M AA solution in its upper two thirds (Fig. 3). Fe^0^ was leached daily for five consecutive days (Monday–Friday) every week with about 180 mL of a 0.02 M ascorbic acid solution (pH = 3.5), at constant temperature of 23 ± 2 °C. At each leaching event, the exact volume of the leachate was monitored and its iron concentration was determined. The experiment was ended after 55 leaching events. This corresponds to a leaching rate of 53% for sponge iron (the most reactive material). An accompanying experiment with 2.0 g of iPuTec was performed to enable the assessment of the impact of the Fe^0^ mass on the extent of Fe leaching by AA.

#### Analytical methods

Analysis for iron was performed using the Phenanthroline method. Although Fe(AA) was already Fe(II), reduction was performed to follow the analytical protocol which include calibrating the standard solutions. Iron concentrations were determined by a Cary 50 UV–Visible Spectrophotometer (Cary instruments, LabMakelaar Benelux B.V., Zevenhuizen, The Netherlands) at a wavelength of 510.0 nm using 1.0 cm glass cells. The pH values were measured by combined glass electrodes (WTW Co., Weinheim, Germany).

### Results and discussion

**Figure 4:**
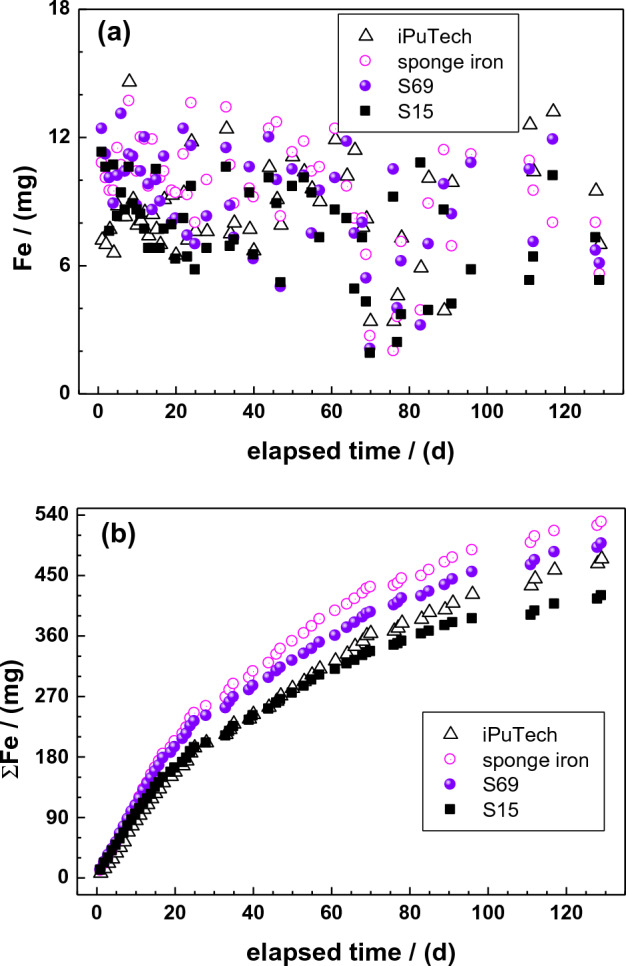
Time-dependent extent of Fe leaching from the four tested Fe^0^ specimens: (**a**) mass per leaching event, and (**b**) cumulative masses. Experimental conditions: m_iron_ = 1.0 g, [AA] = 0.02 M, and T = 23 ± 2 °C.

Table 4 and Fig. 4 summarize the results of Fe extraction from the four tested Fe^0^ materials. It is seen from Table 3 that sponge iron exhibited the highest extent of Fe leaching with 529.5 mg or 53% of the initial 1.0 g after 55 leaching events over 129 days. The increasing order of Fe^0^ reactivity with respect to the extent of Fe leaching in 0.02 M AA is: S15 < iPuTec < S69 < sponge iron. The high reactivity of sponge iron is attributed to its higher porosity and the corresponding surface area in comparison to other materials. The same order of reactivity was reported in related works^58,76^. Another important feature from Table 4 is the fact that using twice the amount of Fe^0^ (2.0 g for iPuTec) did not double the extent of Fe leaching. In fact, when doubling the initial Fe^0^ mass, the daily leached mass of Fe increased by only 24.4%, from 8.6 to 11.4%. This observation is consistent with the non-linear kinetics of Fe^0^ dissolution^57^.

**Table 3:** Elemental composition of iron materials used in this study.

Fe^0^	Element (%)								
	C	Si	Mn	P	S	Cr	Mo	Ni	Fe
Sponge iron	1.96	0.12	0.09	0.027	0.14	0.003	n.d	< 0.001	bal.
iPuTec	3.39	0.41	1.10	n.d	0.105	0.34	n.d	0.088	bal.
S69	3.52	2.12	0.93	n.d	n.d	0.66	n.d	n.d	bal.
S15	0.45	0.28	0.78	n.d	n.d	2.67	n.d	1.34	bal.

*n.d. = not determined and (**) bal. = balance.

**Table 4:** Summary of the extent of Fe leaching from the tested Fe^0^ specimens after 55 leaching events.

Leaching rate		S15	iPuTec	S69	sponge iron	iPuTec (2)
Daily	(mg)	7.6	8.6	9.0	9.6	11.4
Total	(mg)	420	475	497	530	628
Total	(%)	42.0	47.5	49.7	53.0	31.4

The daily leaching is the sum of the leaching mass divided by 55, ‘iPuTec (2)’ corresponds to the experiment using 2.0 g of Fe^0^.

Figure 4a shows that the daily dose of 2–12 mg of Fe could be leached from each column containing 1 g of Fe^0^.

For each material, the leached amount was high at the start of the experiment, then it decreased progressively with increasing leaching events (elapsed time) until about 70 days. It then increased again to values comparable to initial values for all Fe^0^ specimens except S15 until day 110 (Table 5). After day 110, a new decrease of the leached Fe level started. The trend was the same for all Fe^0^ specimens including S15, with only differences in magnitude. Interestingly, around day 70, sponge iron exhibited the lowest extent of Fe leaching. Figure 4b depicts the cumulative extent of Fe leaching and shows clearly that sponge iron is the best material over the 129 leaching events.

**Table 5:** Daily Fe leached mass (mg) from 1.0 g of the tested Fe^0^ specimens at 8 selected events.

Event (−)	Time (d)	IPuTec (mg)	Sponge iron (mg)	S15 (mg)	S69 (mg)
2	2	7.0	10.1	10.6	11.2
10	10	7.9	10.4	8.6	8.8
20	22	9.4	11.2	8.2	12.4
30	44	10.6	12.4	10.1	12.0
40	68	7.8	8.2	7.3	8.0
50	96	13.0	11.2	5.8	10.8
51	111	12.6	10.9	5.3	10.5
52	112	10.4	9.5	6.4	7.1

The Fe^0^ specimens are ordered from left to right in the order of increasing value of m after the second leaching event, corresponding to day 2 of the experiment.

A combination of (1) non-constant kinetics of iron corrosion for individual materials, and (2) different laws of the variation kinetics amount materials, make any prediction of the leaching extent challenging (Table 5). Table 5 shows that for the first 10 leaching events, the increasing order of reactivity was iPuTec < sponge iron < S15 < S69. After this initial period, S15 was the least reactive material until t = 112 d, corresponding to the 52nd leaching event. Between the 10th leaching event and the 52nd there is also no uniform trend in the variation of the extent of Fe leaching from the three other materials. However, it is certain that various amounts of Fe(AA)^2+^ can be obtained to prepare diluted solutions to prevent or combat IDA by varying the following factors: (1) the Fe^0^ mass (e.g. 1.0 g, 2.0 g), (2) the Fe^0^ type (e.g. sponge iron, iPuTec), (3) the AA concentration (e.g. 0.02 M, 0.2 M), and eventually (4) acidifying the solution. Fe^0^ can first be leached by EDTA and the resulting solution (Fe^III^EDTA) reduced and stabilized to Fe(AA)^2+^. In fact, preliminary experiments (results not shown) have demonstrated that EDTA is a far better lixiviant than AA. The ability of AA to reduce Fe^III^EDTA is documented and used in analytical chemistry^29,58^.

This experiment has unequivocally shown that using two columns containing the same amount of a Fe^0^ specimen (m) will yield a larger leaching Fe level than a single column containing 2 times the same materials (2 * m). This is due to extreme complexity of the phenomena associated with aqueous iron corrosion (Table 1)^29,57^. Summarized, these results prove that Fe^0^ leaching using AA is a promising approach to generate stable Fe^II^ solutions to improve the iron status of humans.

## Designing a Fe^II^-bearing unit

**Figure 5:**
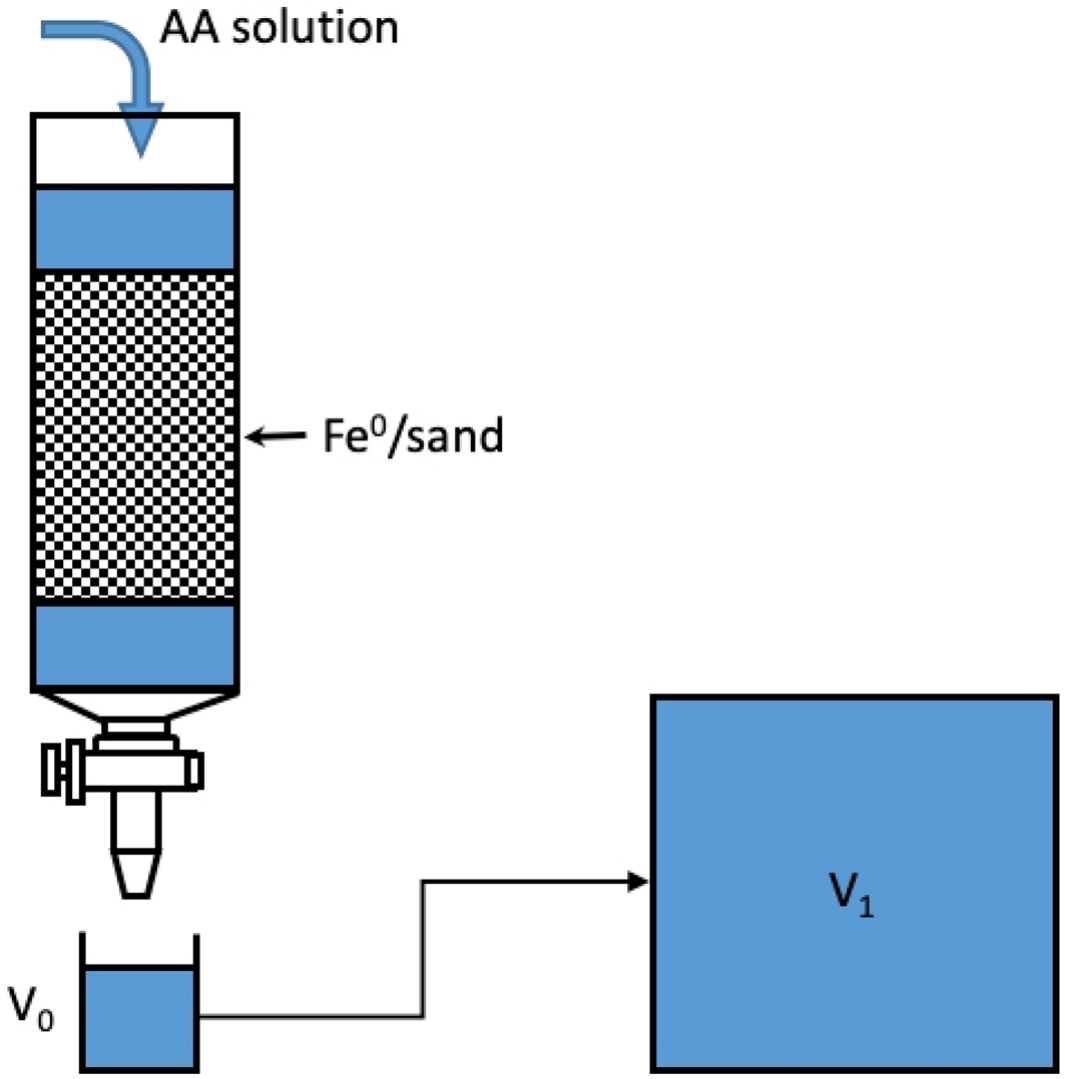
Schematic representation of the process of generating the Fe^II^-AA solution (V_0_) and adding it to a safe drinking water storage tank (V_1_). Fe^0^ is a reactive iron source. Sand is used as a filling material.

The conceptual design of a Fe^II^-AA production unit involves two components: (1) a reactive source of metallic iron (Fe^0^), and (2) an ascorbic acid (AA) solution. In principle, batch and column leaching operations are possible. However, column operations are preferred herein mainly because they can run for several months with limited labor input ("Fe0^0^ leaching with ascorbic acid: proof of concept" section). "Fe^0^ leaching with ascorbic acid: proof of concept" section and available data on Fe^0^ leaching by ethylenediaminetetraacetic acid (EDTA)^29^ suggest that it is possible to leach constant amounts of Fe from Fe^0^ filling, sponge iron, and steel wool placed in a glass column for several weeks^77^ (Fig. 5). The Fe concentration in the effluent (C_0_) depends mainly on the intrinsic reactivity of used Fe^0^, the Fe^0^ mass used, the flow velocity of the AA solution, and the AA concentration. The C_0_ value (Eq. 5) is selected such that a certain volume of the effluent (V_0_) is added to a water reservoir (V_1_) to obtain the desired concentration of Fe in the fortified drinking water (C_1_).

5 $${\text{C}}_{0} = {\text{C}}_{{1}} *{\text{V}}_{{1}} /{\text{V}}_{0}$$

Assuming that available safe drinking water is iron free, the mass balance of Fe implies that C_0_V_0_ = C_1_V_1_ (Eq. 1). If 1 m^3^ Fe^II^-AA fortified water (V_1_ = 1000 L) containing 2 mg/L Fe^II^ (C_1_) is to be daily produced, and 1.0 L (V_0_) of the effluent should be used, then the C_0_ value should be 2000 mg/L or 2.0 g/L (C_0_ = 2000 mg/L). The challenge is to find the best combination of Fe^0^ materials (e.g. iron filings, sponge iron), Fe^0^ mass, AA concentration, and flow velocity of the AA solution, yielding 1 L of a 2.0 g/L Fe^II^ effluent. In case 2.0 g/L Fe is not realistic, one should rather seek to have 10 L of the effluent with 0.2 g/L Fe^II^ (C_0_ = 200 mg/L).

Figure 5 shows an operational device for the production of the Fe^II^-AA effluent for dilution. For the realization of this concept, common affordable laboratory devices for weighing (Fe^0^, AA) and analytically determining Fe are needed. This means that for the development of the Fe^II^-AA method, a small chemical laboratory or Fe^II^ sensor is necessary. However, once the method is established, a laboratory is no longer necessary, and trained personnel can build columns to leach Fe^0^ and perform dilution in water tanks. Calculations are made herein for 1 m^3^. For larger populations, the 1 m^3^ water device can be used as a module, and as many modules as necessary can be used to cover the needs. The operational C_1_ value of 2 mg/L is purely arbitrarily considered. More relevant values should be selected for testing.

A survey of the literature reveals that various Fe doses have been administered to persons in individual studies. For example, Ginanjar et al.^25^ discussed the results of some previous studies using oral supplements of a 0 mg (placebo) to 100 mg (therapy) Fe dose in 200 mL water. Fe was added either as FeSO_4_ or NaFeEDTA and was administered to test persons after at least eight hours of fasting. In other words, up to 100 mg of Fe represents the daily dose to prevent and/or cure IDA. On the other hand, Rakanita et al.^13^ reported that women need 30–60 mg Fe/day. The World Health Organization recommends up to 30 mg Fe/day for children under five^11,54^. Table 6 summarizes the masses of FeSO_4_, Fe^II^ fumarate, Fe^II^ gluconate, and NaFeEDTA necessary to obtain 1 kg of elemental iron (Fe). It is seen that (1) 3.0 to 8.0 kg of salts are needed where just 1 kg of Fe^0^ suffices, (2) FeSO_4_ is more than 50 times the price of iron nails (Fe^0^). However, the (bio)availability of Fe from Fe^0^ is primarily uncertain. To design an appropriate Fe^II^-AA production unit, Eq. 5 is used. The system is operated such that three liters of the drinking water (C_1_) bring the needed daily Fe dose for IDA prevention. For curative issues, (up to 100 mg/d), appropriate designs can be developed on the same basis.

**Table 6:** Common iron salts used for food and water fortification and their corresponding mass to obtain 1 kg of elemental iron.

Iron source	Formula	M (g/mol)	x (−)	m_source_ (kg)	Price (Eur/kg)
Elemental iron	Fe	56.0	1.00	1.00	3.00
Fe(II) fumarate	C_4_H_2_FeO_4_	170.0	0.33	3.03	95.80
Fe(II) sulfate	FeSO_4_.7H_2_O	278.0	0.20	4.96	163.40
NaFeEDTA	C_10_H_12_FeN_2_NaO_8_	367.0	0.15	6.55	299.00
Fe(II) gluconate	C_12_H_24_FeO_14_	448.0	0.12	8.00	102.00

‘x (−)’ is the mass ratio of Fe in the salt. The given kg prices of the salts (chemicals) are from Fisher Scientific (https://www.fishersci.com/—Accesses 26/09/2022). The given Fe^0^ price corresponds to commercial iron nails^78^.

Fe leaching as used herein is extensively employed in extractive metallurgy and in reclamation of mining media^79–82^. The operational parameters impacting the effectiveness of the leaching process include concentration of the AA solution, duration of the leaching operation (long-term corrosion rate), Fe^0^ grain size, Fe^0^ intrinsic reactivity, flow velocity of the AA solution (contact time), and leaching temperature. Given that the kinetics of iron corrosion are neither constant nor linear (see "Fe^0^ leaching with ascorbic acid: proof of concept" Section)^29,82–84^ the service life of each Fe^II^-AA production unit (Fig. 5) cannot be predetermined. In other words, the question on when to recharge a Fe^0^/sand column with fresh Fe^0^ can only be answered by testing.

## IDA and safe drinking water provision: killing two birds with one stone?

The presentation until here has revealed that many low-income settings are still seeking for reliable ways out of the iron deficiency crisis. Past Fe^0^-based attempts to overcome this problem include: (1) using iron cookwares, (2) adding iron ingots while cooking with aluminum cookwares, and (3) consuming food fortified with Fe^0^ powders. The latter is not suitable because of limited access to commercial fortified foods especially for low-income and vulnerable households. All three tools suffer from the natural time-dependent decrease of the kinetics of iron corrosion (decreased corrosion rate or “reactivity loss”)^29,82–87^. On the other hand, limited access to medical care and other costly iron supplements make other available tools for improving iron status less suitable for generalized use in low-income communities.

During the past three decades, a substantial body of evidence has demonstrated that iron intake from drinking water is a powerful weapon against IDA^7,31,39^. In this context, Fe^II^ is either naturally available, for example from groundwater^30,31,34,35^, or artificially added, for example as ferrous sulfate (FeSO_4_)^21^. FeSO_4_ is reported to be the best water-soluble and cheapest iron salt available (Table 6)^7^. Dutra-de-Oliveira et al.^21^ used 10 mg of FeSO_4_ and 100 mg of ascorbic acid (AA) per litre of drinking water. 10 mg of FeSO_4_ contains 3.7 mg of Fe, 2.1 mg of S and 4.2 mg of O. This implies that just 3.7 mg Fe is needed for 1 L or some 4.0 g for 1 m^3^ of water. In other words, 1 kg of Fe^0^ will produce more than 250 m^3^ of Fe fortified drinking water. The price of 1 kg of Fe^0^ (3.00 Euro)^78^ is far less than that of 1 kg of FeSO_4_ (Table 6), and Fe^0^ is readily available, for instance as iron nails or sponge iron^77,88,89^. The advantage of water as a vehicle for Fe is summarized by Dutra-de-Oliveira et al.^7^ as follows: “Water is consumed daily, everywhere by all ages”, including children, pregnant women, and adults of all ages. In other words, Dutra-de-Oliveira et al.^7,90,91^ have already demonstrated the success of iron-fortified drinking water to improve the iron status of low-income populations mainly consuming low iron (Fe^II^) vegetable diet and daily drinking local water^90–96^. Consequently, provided local water is of drinking quality, a universal solution to defeat IDA is made more accessible and affordable by using the Fe^II^-AA method presented herein ("Current use of Fe^0^ for IDA control" section). AA for Fe^0^ leaching is readily, commercially available. For example, in July 2023, 2.5 kg ascorbic acid (vitamin C), food grade can be purchased from Amazon Germany (www.amazon.de) for just 33 Euro.

The past two decades have witnessed the development of affordable solutions for safe drinking supply^97–102^. From these technologies, one is based on filtration on Fe^0^/sand beds^97,100,103–107^. In principle, it is possible to design a Fe^0^ filter capable of releasing about 2 mg/L Fe^II^ in the effluent. In such a case, it suffices to add a diluted solution of ascorbic acid to stabilize Fe^II^ and make it available to the human body. Research is needed to achieve the proverbial notion of “killing two birds with one stone”: (1) safe drinking water, and (2) iron-fortified water, in a decentralized manner. The problem of clean drinking water supply and IDA co-occur or are juxtaposed in low-income countries^108^. This points to the novelty of coupling clean drinking water supply based on Fe^0^ filter systems to the fortification of drinking water to overcome IDA.

## Concluding remarks

There are three main approaches to control IDA: (1) supplementation with iron and folic acid tablets, (2) fortification with iron salts, metal iron and dissolved iron, and (3) natural food-based approaches. Efforts for wide implementation of the first two approaches have not really been successful in combating IDA over the past three decades^14,15,109–112^. The third approach is attractive as it focuses on dietary diversification and enrichment of diets with naturally iron-rich foods, but it is difficult to bring it to scale. Thus, more affordable and applicable tools are still needed.

The Fe^II^-AA approach is an improved version of a 30-year-old method using commercially available highly soluble Fe^II^ salts^7^. Home iron fortification of water supplies bioavailable iron to rural and urban populations and is optimal for mass supply in schools and other institutions. Systematic research is needed to develop scalable Fe^II^-AA producing units. Well-designed experiments are needed to determine the practicality of several potential Fe^0^ materials to serve as reliable Fe sources and to combat IDA.

### Supplementary Information

Supplementary Tables.

## Data Availability

All data generated or analyzed during this study are included in this published article and its supplementary information files.

## References

[1] Dewey KG, Adu-Afarwuah S (2008). Systematic review of the efficacy and effectiveness of complementary feeding interventions in developing countries. Matern. Child Nutr..

[2] Martins JM (2012). Universal iron fortification of foods: the view of a hematologist. Rev. Bras. Hematol. Hemoter..

[3] Stelle I, Kalea AZ, Pereira DIA (2019). Iron deficiency anaemia: experiences and challenges. Proc. Nutr Soc..

[4] Kumari A, Chauhan AK (2022). Iron nanoparticles as a promising compound for food fortification in iron deficiency anemia: A review. J. Food Sci. Technol..

[5] Field MS, Mithra P, Estevez D, Peña-Rosas JP (2020). Wheat flour fortification with iron for reducing anaemia and improving iron status in populations. Cochrane Database Syst. Rev..

[6] Miller JL (2013). Iron deficiency anemia: a common and curable disease. Cold Spring Harb. Perspect. Med..

[7] Dutra-de-Oliveira JD, Marchini JS, Lamounier JA, de Almeida CAN (2016). A new family home approach to controlling iron deficiency anemia in all ages in less-developed and developing countries using iron-fortified water. Arch. Latin Nutr..

[8] Cappellini MD, Musallam KM, Taher AT (2020). Iron deficiency anaemia revisited. J. Int. Med..

[9] Horton S, Ross J (2007). The economics of iron deficiency. Food Pol..

[10] WHO (2014). Global Nutrition Targets 2025 Anaemia Policy Brief (WHO/NMH/NHD/14·4).

[11] WHO (2016). Guideline: Daily Iron Supplementation in Adult Women and Adolescent Girls.

[12] Beinner, M. A. Fortification of drinking water with iron and ascorbic acid in eight municipal day-care centers in Brazil. Doctoral dissertation, (University of Brasilia, Distrito Federal, 2002)

[13] Rakanita Y, Sinuraya RK, Suradji EW, Suwantika AA, Syamsunarno MRAA, Abdulah R (2020). The challenges in eradication of iron deficiency anemia in developing countries. Sys. Rev. Pharm..

[14] Hurrell RF (2021). Iron fortification practices and implications for iron addition to salt. J. Nutr..

[15] Hurrell RF (2021). The potential of iodine and iron double-fortified salt compared with iron-fortified staple foods to increase population iron status. J Nutr..

[16] Shields A, Ansari MA (2021). Review of experience of the production of salt fortified with iron and iodine. J Nutr..

[17] Lermyte F, Zhang W-Y, Brooks J, Huband S, Collingwood JF, Lees MR (2020). Metallic iron in cornflakes. Food Funct..

[18] Tripp K, MacKeith N, Woodruff B, Talley L, Mselle L, Mirghani Z (2010). Acceptability and use of iron and iron-alloy cooking pots: Implications for anaemia control programmes. Public Health Nutr..

[19] Arcanjo FPN, Arcanjo CC, Santos PR (2019). Iron pots for the treatment of iron deficiency anemia: Is there sufficient favorable evidence?. Nutr Food Sci Int J..

[20] Arcanjo FPN, dos Anjos-Sena IV, Queiroz-de-Oliveira AF, Duarte-dos-Santos M, Chagas-de-Cavalcante JC (2020). Iron pots for the treatment of iron deficiency anemia: Is there enough favorable evidence?. Int. J. Health Sci..

[21] Dutra de Oliveira JE, Ferreira JF, Vasconcellos VP, Marchini JS (1994). Drinking water as an iron canier to control anemia in preschool children in a day-care center. J. Am. Coll. Nutr..

[22] Dutra de Oliveira JEE, Amaral-Scheid MM, Desai ID, Marchini S (1996). Iron fortification of domestic drinking water to prevent anemia among low socioeconomic families in Brazil. Int. J. Food Sci. Nutr..

[23] Dutra-de-Oliveira JE, de Almeida CAN (2002). Domestic drinking water—an effective way to prevent anemia among low socioeconomic families in Brazil. Food Nutr. Bull..

[24] Dutra-de-Oliveira JE, Marchini JS, Lamounier J, Almeida CAN (2011). Iron-fortified drinking water studies for the prevention of children's anemia in developing countries. Anemia.

[25] Ginanjar E, Indrawati L, Setianingsih I, Atmakusumah D, Harahap A, Timan IS (2018). Iron absorption in iron-deficient women, who received 65 mg Fe with an Indonesian breakfast, is much better from NaFe(III)EDTA than from Fe(II)SO_4_, with an acceptable increase of plasma NTBI. A randomized clinical Trial. Pharmaceuticals.

[26] de Almeida CAN, Dutra-de-Oliveira JE, Crott GC, Cantolini A, Ricco RG, Del Ciampo LA (2005). Effect of fortification of drinking water with iron plus ascorbic acid or with ascorbic acid alone on hemoglobin values and anthropometric indicators in preschool children in day-care centers in Southeast Brazil. Food Nutr. Bull..

[27] Hsieh YHP, Hsieh YP (1997). Valence state of iron in the presence of ascorbic acid and ethylenediaminetetraacetic acid. J. Agric. Food Chem..

[28] Mehansho H (2006). Iron fortification technology development: New approaches. J. Nutr..

[29] Lufingo M, Ndé-Tchoupé AI, Hu R, Njau KN, Noubactep C (2019). A novel and facile method to characterize the suitability of metallic iron for water treatment. Water.

[30] Li N, Zhao G, Wu W, Zhang M, Liu W, Chen Q (2020). Wang X The efficacy and safety of vitamin C for iron supplementation in adult patients with iron deficiency anemia: A randomized clinical trial. JAMA Netw. Open..

[31] Pasupathy E, Kandasamy R, Thomas K, Basheer A (1818). Alternate day versus daily oral iron for treatment of iron deficiency anemia: A randomized controlled trial. Sci Rep.

[32] Briend A, Hoque B, Aziz K (1990). Iron in tubewell water and linear growth in rural Bangladesh. Arch. Dis. Child..

[33] Merrill RD, Shamim AA, Ali H, Jahan N, Labrique AB, Schulze K (2011). Iron status of women is associated with the iron concentration of potable groundwater in rural Bangladesh. J Nutr..

[34] Merrill R (2012). Iron in groundwater: A source for anemia prevention. Vitam. Trace Elem..

[35] Merrill RD, Shamim AA, Ali H, Labrique AB, Schulze K, Christian P, West KP (2012). High prevalence of anemia with lack of iron deficiency among women in rural Bangladesh: Arole for thalassemia and iron in groundwater. Asia Pac. J. Clin. Nutr..

[36] Karakochuk CD, Murphy HM, Whitfield KC, Barr SI, Vercauteren SM, Talukder A (2015). Elevated levels of iron in groundwater in Prey Veng province in Cambodia: A possible factor contributing to high iron stores in women. J. Water Health..

[37] Wendt AS, Waid JL, Gabrysch S (2019). Dietary factors moderate the relation between groundwater iron and anemia in women and children in rural Bangladesh. Curr. Dev. Nutr..

[38] Rahman S, Kortman GAM, Boekhorst J, Lee P, Khan MR, Ahmed F (2021). Effect of low-iron micronutrient powder (MNP) on the composition of gut microbiota of Bangladeshi children in a high-iron groundwater setting: Arandomized controlled trial. Eur. J. Nutr..

[39] Langmuir D (1997). Aqueous Environmental Geochemistry.

[40] Wheeler WE. Solubility and bioavailability of metallic iron in drinking water. Master Dissertation, 60 (University of California, Davis, 1994).

[41] Mohammadi M, Khashayar P, Tabari M, Sohrabvandi S, Moghaddam AF (2016). Water fortified with minerals (Ca, Mg, Fe, Zn). Int. J. Med. Res. Health Sci..

[42] González JIV, González DF, González LFV (2020). Operations and Basic Processes in Ironmaking.

[43] Matsukevich I, Kulinich N, Romanovski V (2022). Direct reduced iron and zinc recovery from electric arc furnace dust. J. Chem. Technol. Biotechnol..

[44] Charles, C. V. Happy Fish: a novel supplementation technique to prevent iron deficiency Anemia in women in rural Cambodia. PhD Dissertation, (The University of Guelph, 2012).

[45] Gordeuk VR, Boyd DR, Brittenham GM (1986). Dietary iron overload persists in rural sub-Saharan Africa. Lancet.

[46] Walker ARP, Segal I (1999). Iron overload in Sub-Saharan Africa: To what extent is it a public health problem?. Br. J. Nutr..

[47] Andrews NC (2000). Iron metabolism: Iron deficiency and iron overload. Ann. Rev. Genom. Hum. Genet..

[48] Charles CV, Dewey CE, Daniell WE, Summerlee AJ (2010). Iron-deficiency anaemia in rural Cambodia: Community trial of a novel iron supplementation technique. Eur. J. Public Health..

[49] Charles CV, Dewey CE, Hall A, Hak C, Channary S, Summerlee AJ (2015). A randomized control trial using a fish-shaped iron ingot for the amelioration of iron deficiency anemia in rural Cambodian women. Trop. Med. Surg..

[50] Charles CV, Summerlee AJ, Dewey CE (2011). Iron content of Cambodian foods when prepared in cooking pots containing an iron ingot. Trop. Med. Int. Health..

[51] Borigato EV, Martinez FE (1998). Iron nutritional status is improved in Brazilian preterm infants fed food cooked in iron pots. J. Nutr..

[52] Alves C, Saleh A, Alaofè H (2019). Iron-containing cookware for the reduction of iron deficiency anemia among children and females of reproductive age in low- and middle-income countries: A systematic review. PLoS ONE.

[53] Rappaport AI, Whitfield KC, Chapman GE, Yada RY, Kheang KM, Louise J (2017). Randomized controlled trial assessing the efficacy of a reusable fish-shaped iron ingot to increase haemoglobin concentration in anemic, rural Cambodian women. Am. J. Clin. Nutr..

[54] Rodriguez-Ramiro I, Perfecto A, Fairweather-Tait SJ (2017). Dietary factors modulate iron uptake in Caco-2 cells from an iron ingot used as a home fortificant to prevent iron deficiency. Nutrients.

[55] Ebert, C., Heesemann, E., & Vollmer S. Two interventions to promote health and mental development in early childhood: A randomized controlled trial in rural India. Discussion Paper 276, (Courant Research Centre 'Poverty, Equity and Growth', Georg-August-Universität Göttingen, 2021).

[56] Krämer M, Kumar S, Vollmer S (2021). Improving child health and cognition: Evidence from a school-based nutrition intervention in India. Rev. Econ. Stat..

[57] Yang H, Liu Q, Hu R, Ptak T, Taherdangkoo R, Noubactep C (2022). Numerical study on long-term effectiveness of metallic iron based permeable reactive barriers: Importance of porosity heterogeneity of the barrier. J. Hydrol..

[58] Cui X, Xiao M, Tao R, Hu R, Ruppert H, Gwenzi W, Noubactep C (1930). Developing the ascorbic acid test: A candidate standard tool for characterizing the intrinsic reactivity of metallic iron for water remediation. Water.

[59] Hurrell R, Bothwell T, Cook JD, Dary O, Davidsson L, Fairweather-Tait S (2002). The usefulness of elemental iron for cereal flour fortification: A Sustain task force report. Nutr. Rev..

[60] Kew MC, Asare GA (2007). Dietary iron overload in the African and hepatocellular carcinoma. Liver Inter..

[61] Armstrong GR, Dewey CE, Summerlee AJS (2017). Iron release from the Lucky Iron Fish®: Safety considerations. Asia Pac. J. Clin. Nutr..

[62] Hurrell RF, Cook JD (1990). Strategies for iron fortification of foods. Trends Food Sci. Technol..

[63] Larsen O, Postma D, Jakobsen R (2006). The reactivity of iron oxides towards reductive dissolution with ascorbic acid in a shallow sandy aquifer (Rømø, Denmark). Geochim. Cosmochim. Acta.

[64] Lahiri A (2010). Influence of ascorbate and oxalic acid for the removal of iron and alkali from alkali roasted ilmenite to produce synthetic rutile. Ind. Eng. Chem. Res..

[65] Sinha MK, Purcell W (2019). Reducing agents in the leaching of manganese ores: A comprehensive review. Hydrometallurgy.

[66] Sinha MK, Purcell W, van Der Westhuizen WA (2020). Recovery of manganese from ferruginous manganese ore using ascorbic acid as reducing agent. Miner. Eng..

[67] Xu P, Wang L, Liu X, Xie S, Yang Z, Zhu P (2022). Ascorbic acid enhanced the zero-valent iron/peroxymonosulfate oxidation: Simultaneous chelating and reducing. Sep. Purif. Technol..

[68] Gasim MF, Bao Y, Elgarahy AM, Osman AI, Al-Muhtaseb AH, Rooney DW, Yap P-S, Oh W-D (2023). Peracetic acid activation using heterogeneous catalysts for environmental decontamination: A review. Catal. Comm..

[69] Xu P, Wang L, Liu X, Xie S, Hou B (2023). Vitamin C promoted refractory organic contaminant elimination in the zero-valent iron/peracetic acid system: Efficiency, mechanism and effects of various parameters. Chemosphere.

[70] Zhu S, Zhang Y, Zhang Z, Ai F, Zhang H, Li Y, Wang Y, Zhang Q (2023). Ascorbic acid-mediated zero-valent iron enhanced hydrogen production potential of bean dregs and corn stover by photo fermentation. Bioresour. Technol..

[71] Conrad ME, Schade SG (1968). Ascorbic acid chelates in iron absorption: A role for hydrochloric acid and bile. Gastroenterology.

[72] Teucher B, Olivares M, Cori H (2004). Enhancers of iron absorption: ascorbic acid and other organic acids. Int. J. Vitam. Nutr. Res..

[73] Elomaa H, Seisko S, Lehtola J, Lundström M (2019). A study on selective leaching of heavy metals vs. iron from fly ash. J. Mater. Cycles Waste Manag..

[74] Chen D, Rao S, Wang D, Cao H, Xie W, Liu Z (2020). Synergistic leaching of valuable metals from spent Li-ion batteries using sulfuric acid- l-ascorbic acid system. Chem. Eng. J..

[75] Du K, Ang EH, Wu X, Liu Y (2022). Progresses in sustainable recycling technology of spent lithium ion batteries. Energy Environ. Mater..

[76] Ndé-Tchoupé AI, Hu R, Gwenzi W, Nassi A, Noubactep C (2020). Characterizing the reactivity of metallic iron for water treatment: H_2_ evolution in H_2_SO_4_ and uranium removal efficiency. Water.

[77] Dorey C, Cooper C, Dickson DPE, Gibson JF, Simpson RJ, Peters TJ (1993). Iron speciation at physiological pH in media containing ascorbate and oxygen. Br. J. Nutr..

[78] Hildebrant B, Ndé-Tchoupé AI, Lufingo M, Licha T, Noubactep C (2020). Steel wool for water treatment: Intrinsic reactivity and defluoridation efficiency. Processes.

[79] dos Santos NO, Teixeira LA, Zhou Q, Burke G, Campos LC (2022). Fenton pre-oxidation of natural organic matter in drinking water treatment through the application of iron nails. Environ. Technol..

[80] Pierce EM, Wellman DM, Lodge AM, Rodriguez EA (2007). Experimental determination of the dissolution kinetics of zero-valent iron in the presence of organic complexants. Environ. Chem..

[81] Han L, Chen B, Liu T, Choi Y (2019). Leaching characteristics of iron and manganese from steel slag with repetitive replenishment of leachate. KSCE J. Civ. Eng..

[82] Jie YJ, Kamaruddin S, Mustapha M, Siddiquee AN, Al-Ahmari A, Khan ZA (2019). Reclamation of steel shots by acid leaching for powder metallurgy applications. Adv. Mechan. Eng..

[83] Alcántara J, de la Fuente D, Chico B, Simancas J, Díaz I, Morcillo M (2017). Marine atmospheric corrosion of carbon steel: A review. Materials..

[84] Stefanoni M, Angst U, Elsener B (2018). Electrochemistry and capillary condensation theory reveal the mechanism of corrosion in dense porous media. Sci. Rep..

[85] Ali N, Fulazzaky MA (2020). The empirical prediction of weight change and corrosion rate of low-carbon steel. Heliyon..

[86] Chen Q, Fan G, Na W, Liu J, Cui J, Li H (2019). Past, present, and future of groundwater remediation research: A scientometric analysis. Int. J. Environ. Res. Public Health.

[87] Li X, Li Z, Du C, Tian Z, Zhu Q, Li G (2021). Bibliometric analysis of zerovalent iron particles research for environmental remediation from 2000 to 2019. Environ. Sci. Pollut. Res..

[88] Yang H, Hu R, Ruppert H, Noubactep C (2021). Modeling porosity loss in Fe^0^-based permeable reactive barriers with Faraday’s law. Sci. Rep..

[89] Noubactep C, Schöner A, Woafo P (2009). Metallic iron filters for universal access to safe drinking water. Clean Soil Air Water.

[90] Dutra-de-Oliveira JE, Marchini JS, Desai I (1996). Fortification of drinking water with iron: A new strategy for combating iron deficiency in Brazil. Amer. J. Clin. Nutr..

[91] Viteri FE (1996). Reply to J.E Dutra-de-Oliveira Amer et al. J. Clin. Nutr..

[92] Dutra-de-Oliveira JE, Marchini JS (2006). Drinking water as an iron carrier to control iron deficiency. Nutrition.

[93] Lamounier JA, Capanema FD, Rocha DS, Dutra-de-Oliveira JE, da Silva MC, Nogueira-de-Almeida CA (2010). Iron fortification strategies for the control of childhood anemia in Brazil. J. Trop. Ped..

[94] Dutra-de-Oliveira JE, Marchini JS, Lamounier J, Nogueira-de-Almeida CA (2012). A community public health programme to control iron-deficiency anemia through iron-fortification of drinking water. Int. J. Nutrol..

[95] Lamounier JA, Capanema FD, Rocha DS, Maddock J (2012). Iron food fortification for the control of childhood anemia in Brazil. Public Health-Social and Behavioral Health.

[96] Nogueira-de-Almeida CA, da Veiga UF, Del Ciampo LA, Martinez EZ, Ferraz IS, Contini AA, Soares da Cruz FC, Silva RFB, Nogueira-de-Almeida ME, Lamounier JA (2021). Prevalence of childhood anaemia in Brazil: Still a serious health problem: A systematic review and meta-analysis. Public Health Nutr..

[97] Tepong-Tsindé R, Crane R, Noubactep C, Nassi A, Ruppert H (2015). Testing metallic iron filtration systems for decentralized water treatment at pilot scale. Water.

[98] Goncharuk VV (2008). A new concept of supplying the population with a quality drinking water. J. Water Chem. Technol..

[99] Domènech L (2011). Rethinking water management: From centralised to decentralised water supply and sanitation models. Doc. An. Geogr..

[100] Siwila S, Brink IC (2018). A small-scale low-cost water treatment system for removal of selected heavy metals, bacteria and particles. Water Pract. Technol..

[101] Huang Z, Nya EL, Cao V, Gwenzi W, Rahman MA, Noubactep C (2021). Universal access to safe drinking water: Escaping the traps of non-frugal technologies. Sustainability.

[102] Kearns J, Dickenson E, Aung MT, Joseph SM, Summers SR, Knappe D (2021). Biochar water treatment for control of organic micropollutants with UVA surrogate monitoring. Environ. Eng. Sci..

[103] Naseri E, Ndé-Tchoupé AI, Mwakabona HT, Nanseu-Njiki CP, Noubactep C, Njau KN, Wydra KD (2017). Making Fe^0^-based filters a universal solution for safe drinking water provision. Sustainability..

[104] Nya EL, Feumba R, Fotsing-Kwetché PR, Gwenzi W, Noubactep C (2021). A hybrid model for achieving universal safe drinking water in the medium-sized city of Bangangté (Cameroon). Water.

[105] Yang H, Hu R, Ndé-Tchoupé AI, Gwenzi W, Ruppert H, Noubactep C (2020). Designing the next generation of Fe^0^-based filters for decentralized safe drinking water treatment. Processes..

[106] Tepong-Tsindé R (2021). Designing and piloting a household filter for the peri-urban population of Douala (Cameroon). Freiberg. Online Geosci..

[107] Lan LE, Reina FD, De Seta GE, Meichtry JM, Litter MI (2023). Comparison between different technologies (zerovalent iron, coagulation-flocculation, adsorption) for arsenic treatment at high concentrations. Water.

[108] Choudhury N, Siddiqua TJ, Tanvir-Ahmed SM, Haque MDA, Ali M, Farzana FD, Naz F, Rahman SS, Faruque ASG, Rahman S, Ahmed T (2022). Iron content of drinking water is associated with anaemia status among children in high groundwater iron areas in Bangladesh. Trop. Med. Int. Health.

[109] Wuehler SE, Hess SY, Brown KH (2011). Accelerating improvements in nutritional and health status of young children in the Sahel region of Sub-Saharan Africa: Review of international guidelines on infant and young child feeding and nutrition. Mater. Child Nutr..

[110] Wieringa FT, Dahl M, Chamnan C, Poirot E, Kuong K, Sophonneary P (2016). The high prevalence of anemia in Cambodian children and women cannot be satisfactorily explained by nutritional deficiencies or hemoglobin disorders. Nutrients.

[111] Lemoine A, Tounian P (2020). Childhood anemia and iron deficiency in sub-Saharan Africa–risk factors and prevention: A review. Arch. Pediatr..

[112] Anitha S, Kane-Potaka J, Botha R, Givens DI, Sulaiman NLB, Upadhyay S (2021). Millets can have a major impact on improving iron status, hemoglobin level, and in reducing iron deficiency anemia—A systematic review and meta-analysis. Front. Nutr..

